# Sleep problems in children with autism spectrum disorder in Hong Kong: a cross-sectional study

**DOI:** 10.3389/fpsyt.2023.1088209

**Published:** 2023-04-17

**Authors:** Man Ho Brian Leung, Sze Ting Joanna Ngan, Pak Wing Calvin Cheng, Fong Chun Grace Chan, Wing Chung Chang, Hoi Kei Cheung, Chung Ho, Chi Kei Krystal Lee, Yiu Chung Vincent Tang, Siu Man Corine Wong, Kwok Ling Phyllis Chan

**Affiliations:** ^1^Department of Psychiatry, Queen Mary Hospital, Hong Kong, Hong Kong SAR, China; ^2^Department of Psychiatry, The University of Hong Kong, Hong Kong, China; ^3^School of Public Health, The University of Hong Kong, Hong Kong, China

**Keywords:** cross-sectional, neurodevelopmental disorder, sleep habits, sleep disorders, autism spectrum disorder

## Abstract

**Background:**

Autism spectrum disorder (ASD) is a neurodevelopmental disorder with a growing prevalence of sleep problems associated with significant behavioral problems and more severe autism clinical presentation. Little is known about the relationships between autism traits and sleep problems in Hong Kong. Therefore, this study aimed to examine whether children with autism have increased sleep problems than non-autistic children in Hong Kong. The secondary objective was to examine the factors associated with sleep problems in an autism clinical sample.

**Methods:**

This cross-sectional study recruited 135 children with autism and 102 with the same age range of non-autistic children, aged between 6 and 12 years. Both groups were screened and compared on their sleep behaviors using the Children's Sleep Habits Questionnaire (CSHQ).

**Results:**

Children with autism had significantly more sleep problems than non-autistic children [*t*_(226.73)_ = 6.20, *p* < 0.001]. Bed -sharing [beta = 0.25, *t*_(165)_ = 2.75, *p* = 0.07] and maternal age at birth [beta = 0.15, *t*_(165)_ = 2.05, *p* = 0.043] were significant factors associated with CSHQ score on the top of autism traits. Stepwise linear regression modeling identified that only separation anxiety disorder (*beta* = 4.83, *t* = 2.40, *p* = 0.019) best-predicted CSHQ.

**Conclusion:**

In summary, autistic children suffered from significantly more sleep problems and co-occurring separation anxiety disorder brings greater sleep problems as compared to non-autistic children. Clinicians should be more aware of sleep problems to provide more effective treatments to children with autism.

## 1. Introduction

Autism spectrum disorder (ASD) is a neurodevelopmental disorder characterized by persistent deficits in social communication and interaction, and restrictive, repetitive patterns of behavior, interest, or activities (1). Individuals with autism were estimated to have a prevalence between 1 and 2% across Asia, Europe, and North America (2). Given the increasing prevalence, autism was associated with significant global and individual burden, as they accounted for 56.3 disability-adjusted life-years per 100,000 population (3). Among patients diagnosed with ASD, 83% were reported to have one or more developmental diagnoses (4).

Various studies have investigated the prevalence and risks of sleep problems among children with autism. Sleep problems were identified in over 70% of the children with autism in a cross-sectional study (5)and the severity of sleep problems seems to be persistent over time. Both objective [i.e., based on actigraphy or polysomnography (PSG)] and subjective (i.e., based on sleep diaries/questionnaires) sleep parameter studies suggested more severe sleep problems in children with autism than in the non-autistic group (6, 7). Hodge et al. revealed that the score of the Children's Sleep Habits Questionnaire (CSHQ), a measure of children's sleep behaviors that may contribute to sleep problems, improved as age increases in the non-autistic group but that was not seen in the autistic group (8). Consistent findings were found by Mazurek et al. who administered the CSHQ to the autism clinical population (9). They reported an initial prevalence of 70% and 65% at follow-up after a mean of 3.8 years and the change was significant (*p* < 0.001), yet 22.9% of the children also reported worsening sleep over time (9).

Limited studies were found in Asia in the area exploring sleep problems with autistic children with a large sampling and standardized tools. In a cross-sectional study in China, bedtime resistance, anxiety, sleep onset delay, and daytime sleepiness were found to be related to the core autism traits (10). Locally in Hong Kong, the prevalence was reported at 16.1 per 10,000 children < 15 years, that is, 1 in 621 children was diagnosed with ASD (11). The prevalence of problem sleepers, defined by at least one problematic sleep behavior in the CSHQ questionnaire was 67.9%, with bedtime resistance (44.6%) being the most common sleep problem (12). It was also highlighted that the practice of regular sleep time and lower parental stress were associated with better sleep quality. In contrast to the findings of Hodge et al. regarding the persistence of sleep problems over time, an increase in age was found to be protective of sleep problems in the local population (8). The differences in the prevalence and risk factors between different cultures suggest an opportunity to understand in-depth findings specific to the culture for tailor-made future interventions and directions.

Sleep disturbance is postulated to be related to the worsening of core autism traits. Gunes et al. reported a positive correlation between CSHQ bedtime resistance (*r* = 0.19, *p* = 0.045) and sleepwalking (*r* = 0.20, *p* = 0.042) subscore and elevated Childhood Autism Rating Scale score (13), signifying more severe autism-related problems (14). Veatch et al. retrieved medical records of 2,714 children with autism from a database to analyze the possible impact of sleep duration on the children and reported that children with short duration of sleep showed an increased social impairment severity on both Autism Diagnostic Interview-Revised (ADI-R) and Autism Diagnostic Observation Schedule (ADOS), both well-validated diagnostic tools for autism, on top of elevated Child Behavior Checklist (CBCL) scores, which detect emotional and behavioral problems in children (13).

Sleep disturbance is also found to correlate with cognitive performance in children with autism. Taylor et al. assessed the cognitive function and sleep disturbances of 335 autistic children. They reported that children who slept more hours per night had higher full scale (*R*^2^ = 0.11, *p* < 0.01) and verbal Intelligent Quotient (IQ) (*R*^2^ = 0.15, *p* < 0.01) (15). Al Backer et al. administered the Cambridge Neuropsychological Test Automated Battery to 18 children with autism and explored its correlation to their CSHQ scores (16). They reported that good sleepers performed significantly better in the simple reaction time task, while bedtime resistance (*r* = 0.531, *p* = 0.023) and sleep anxiety (*r* = 0.474, *p* = 0.047) significantly correlated with the response time of Serial Reaction Time task (16).

Sleep problems in children with autism are postulated to be caused by biopsychosocial factors (17). Neurobiological disruptions in autistic children may have led to disruption of the circadian sleep–wake cycle and the sleep architecture (18, 19). Increased blood levels of serotonin (20) or decreased nocturnal production of melatonin (21) have been found in autism as opposed to healthy individuals that are related to sleep dysregulation and insomnia (22). Medical and psychiatric issues such as co-occurring conditions that cause pain or discomfort or psychiatric conditions such as ADHD, anxiety, and depression were also found to be paramount risk factors for sleep problems in children with autism (23). In addition, obstructive sleep apnea is also an important co-occurring condition that contributed to the suboptimal sleep quality of children with autism (19, 24). Other medical factors that may affect sleep include gastrointestinal difficulties, pain, seizures, asthma, sinusitis, and restless sleep (19). Kose et al. identified risk factors for sleep problems in the autistic population. They identified a 2.8-fold increase in the risk of sleep disturbance in those with neurodevelopmental disorders and a 13.1-fold increase by co-sleeping with parents (25). Higher bedtime resistance and sleep anxiety may have also contributed to the elevated CSHQ scores in children with autism than in non-autistic children (14).

Sleep disturbance is postulated to have a negative impact on the clinical characteristics of autism and associated behavior traits. Children with short duration of sleep showed greater social and emotional impairments (12, 26, 27). The severity of sleep problems was shown to be positively correlated with autism traits and the prevalence of caregivers' mood disorders (28). Multiple studies revealed the impact of sleep disturbance in autistic children was associated with behavioral disturbance (9, 29). Anxiety level and sensory over-responsivity of children were also associated with sleep problems in autistic children (30–32).

To the best of our knowledge, to date, there are limited studies with enough sample size and quality to provide further information on the association between autism characteristics and sleep problems in school-age children in Hong Kong. It is important to conduct culturally sensitive studies taking into consideration of the local environment such as school stress and high-stake testing to better understand and further inform directions specific to the local context. Therefore, the primary objective of this study was to test whether children with autism have increased sleep problems than non-autistic children in Hong Kong. The secondary objective was to examine the factors correlated with sleep problems in an autism clinical sample.

## 2. Methods

### 2.1. Design and subjects

This study was approved by the Institutional Review Board of the University of Hong Kong/Hospital Authority Hong Kong West Cluster (reference: UW 19-290), and all methods were performed in accordance with the relevant guidelines and regulations. It was a cross-sectional study of Chinese children with autism and non-autistic children of the same age range. Subjects with autism were new case referrals between July 2018 and September 2019 to the child and adolescent psychiatry clinic at Queen Mary Hospital in Hong Kong. Informed consent was obtained from a legal guardian or parents during recruitment. The inclusion criteria were as follows: 1. Diagnosis of ASD according to DSM-IV; 2. Aged between 6 and 12 with carer; and 3. Speak and understand Cantonese and write Chinese. Children with intellectual disabilities, personal history of depression, mania, or hypomania, and a history of severe medical illnesses such as epilepsy who required long-term medications were excluded.

Individual structured interviews were conducted by experienced child psychiatrists with the Developmental, Dimensional, and Diagnostic Interview (3Di) (33, 34) and the Diagnostic Interview Schedule for Children-Version 4 (DISC-IV) (35, 36) for the diagnosis of ASD and other psychiatric co-morbidities in the autistic group, respectively. Inter-rater reliability was established by videotaping the diagnostic interviews, then one investigator conducted the diagnostic interview, and another viewed the videotaped interview independently.

The non-autistic children were recruited through convenience sampling in local schools and youth centers. The same inclusion criteria were applied for the non-autistic group except for the presence of ASD diagnosis. Parents were also requested to complete the Chinese-, child- or adolescent versions of the Autism Spectrum Quotient (AQ) (37) to screen for the presence of autism traits. Parents in both groups were asked to complete a parent-report Children's Sleep Habits Questionnaire (CSHQ) to assess the sleep problems of their children. Sociodemographic information including bed and room sharing, academic performance, household income, parental education level, parental employment status, parental mental illness, and marital status were collected in our study. A demographic questionnaire and CSHQ were completed by the children's parents.

### 2.2. Measures

#### 2.2.1. Sleep behavior

The parent-report CSHQ (38) is a 33-item parent-report questionnaire to examine the frequency of sleep behavior in school-aged children over the past 1 week. The validated Chinese version of CSHQ has a high internal consistency Cronbach's alpha coefficient of 0.73 and test–retest reliability coefficient of 0.88 (39).

#### 2.2.2. Diagnosis and symptoms of autism

The 3Di is a computerized parental interview to generate the symptoms and diagnostic profiles for autistic subjects. The concurrent criterion was very good with a mean kappa coefficient of 0.74. A local study using Cantonese and a validated version achieved impressive reliability and validity with a sensitivity of 95% and a specificity of 77% (40).

The Autism Spectrum Quotient-Children's Version (AQ-Child) and Adolescent's Version (AQ-Adol) are parent-report questionnaires that aim to quantify autistic traits in children and adolescents (41, 42). Higher scores correspond to increased symptom severity. The Hong Kong version, AQ-Child and AQ-Adol, was adopted in this study with 150 as the maximum score, cutoff set at 76 for both questionnaires with high sensitivity and specificity (43), which is good for screening out the autistic subjects from non-autistic children.

#### 2.2.3. Co-occurring psychiatric conditions

The parent version of Diagnostic Interview Schedule for Children-Version 4 (DISC-IV) is a structured respondent-based interview to assess the presence of psychiatric diagnoses in children aged between 6 and 17 years, which occurred over the past 12 months and 4 weeks (35) for the autism group. The DISC-IV has been translated and validated in the Hong Kong population with ~70% reliability (36).

### 2.3. Statistical analyses

All data analyses were performed using SPSS, version 25.0 (SPSS Inc., Chicago, Illinois). To compare the autistic group with non-autistic children, the Pearson chi-square test and independent *t*-test were used. We investigated the effects of different variables on the primary outcome (the CSHQ total score) using general linear modeling. Further subgroup analyses were conducted to delineate the effect of different co-occurring psychiatric conditions, measured by DISC-IV, on the CSHQ total score. A *p*-value of < 0.05 was considered significant throughout this study.

## 3. Results

### 3.1. Participant characteristics

**Figure 1:**
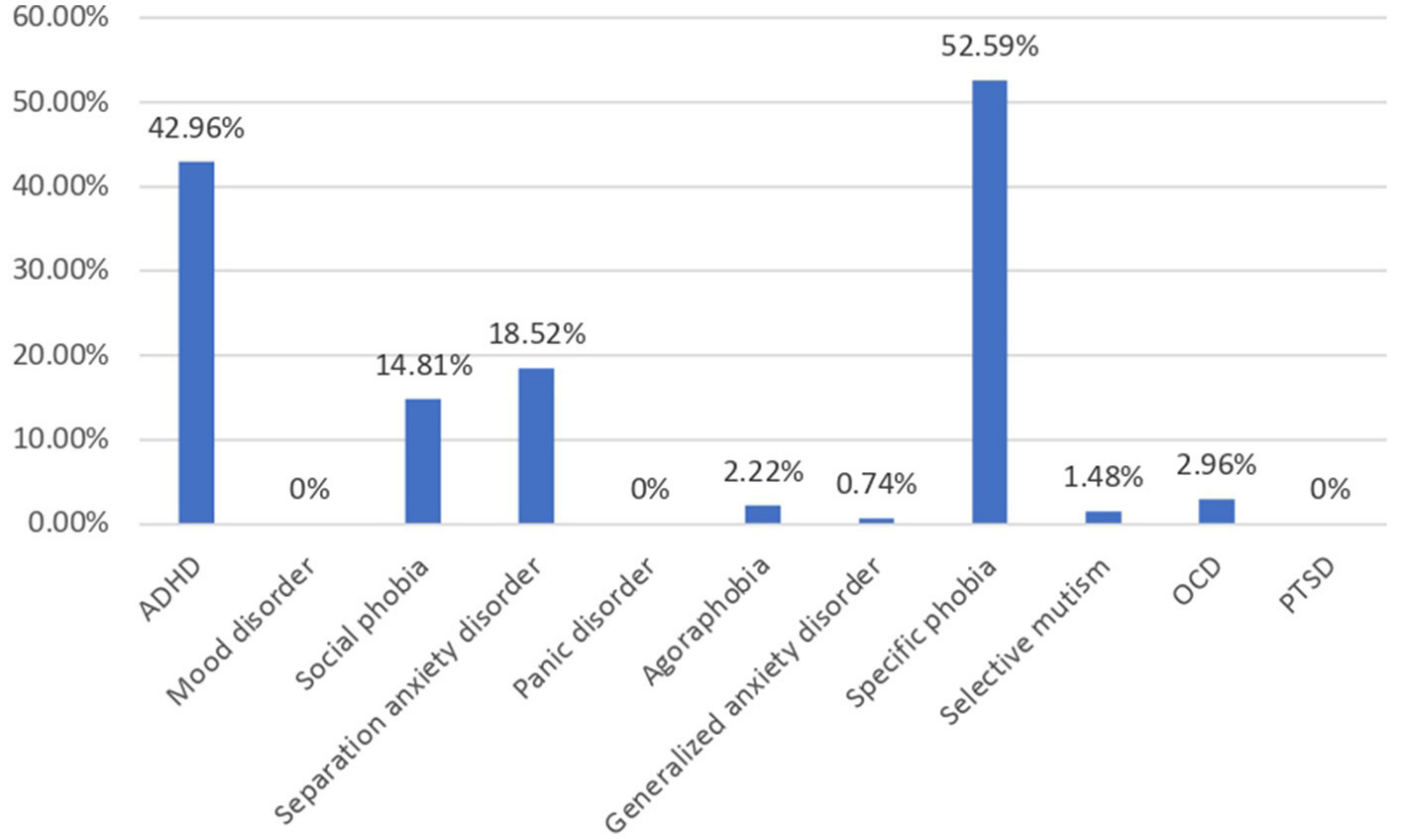
Prevalence of psychiatric comorbidities in austim group (*n* = 135).

A total of 237 children were enrolled in this study, of which there were 135 (56.9%) children with autism and 102 (43.1%) non-autistic children with the same age range as shown in Table 1. The autistic group had a younger mean age (*p* < 0.001), a male-predominant gender ratio (*p* < 0.001), and a higher maternal age (*p* = 0.042) than non-autistic children. The majority of the participants' household income was within 20k-50k, which falls within the median household income at the time of the study in Hong Kong. Approximately 48.1% of parents of autistic children and 46.1% of parents of non-autistic children attained tertiary education, which was higher than 27.3% of the overall population. In total, 30% of parents of autistic children and 13.24% of parents of non-autistic children were unemployed, compared to the overall unemployment rate of 3.5% of the Hong Kong population. Shared beds (*p* < 0.001) and rooms (*p* = 0.019) were more commonly found in autistic than non-autistic children whereas more recently immigrated families (*p* < 0.001) were found in the non-autistic children group. Notably, among the clinical group assessed with DISC-IV, Figure 1 demonstrated the distribution of co-occurring psychiatric conditions among the autistic group. In general, 96 (71.1%) of the children with autism suffered from one or more co-occurring psychiatric conditions, with 58 (43.0%) having co-occurring ADHD and 78 (58.5%) having at least one type of anxiety disorders. Approximately 41 (30.4%) were found to have both co-occurring ADHD and anxiety disorders of any type.

**Table 1:** Characteristics of subjects and their parents in autism and non-autistic group.

	***N* = 237**	**Autism patients *n* = 135 (%)**	**Non-autistic, *n* = 102 (%)**	***t*/Chi-square**	***p***
Mean age		7.01 ± 1.32	8.75 ± 1.98	−7.68	< 0.001^*^
Male	163	113 (83.7)	50 (49.0)	32.55	< 0.001^*^
Female	74	22 (16.3)	52 (51.0)		
School repeater	234	17 (12.6)	5 (4.9)	4.13	0.042^*^
On medications	234	16 (11.9)	3 (2.9)	6.5	0.011^*^
Bed-sharing	200	55 (40.7)	28 (27.5)	14.11	< 0.001^*^
Room-sharing	199	77 (57.0)	61 (59.8)	5.54	0.019^*^
History of child abuse	233	5 (3.7)	0 (0.0)	3.91	0.048^*^
**Family income (HKD/month)**	228			2.07	0.558
< 20 k		25	16		
20 k−50 k		57	40		
50 k−100 k		31	32		
>100 k		14	13		
**Marital status**	234			2.92	0.713
Married		113	81		
Cohabitating		7	8		
Separated		2	3		
Divorced		9	7		
Single		2	0		
Widowed		1	1		
Recent immigrant (< 7 years)	234	6 (2.6)	23 (9.8)	17.63	< 0.001^*^
**Mean parental age at birth**					
Father	217	37.03 ± 5.88	36.46 ± 6.72	0.66	0.518
Mother	218	33.33 ± 5.46	32.00 ± 4.16	2.04	0.042^*^
**Parental education level (tertiary or above)**					
Father	231	70 (30.3)	48 (20.8)	0.75	0.688
Mother	230	60 (26.1)	46 (20.0)	0.25	0.881
**Parental history of mental illness**					
Father	232	9 (3.9)	2 (0.9)	2.83	0.092
Mother	233	23 (9.9)	2 (0.9)	13.63	< 0.001^*^

^*^p < 0.05.

### 3.2. Prevalence of sleep problems

Children with autism have significantly more severe CSHQ total scores compared to the non-autistic children group [*t*_(226.73)_ = 6.20, *p* < 0.001). Using the CSHQ cutoff score of 41, 98 (72.6%) of the autistic children group and 51 (50%) of the non-autistic group had clinically significant sleep problems, which had a significant group difference (χ^2^ = 13.43, *p* < 0.001). Children with autism also have significantly higher scores across all eight CSHQ subscales (*p* < 0.05) except sleep duration (*p* = 0.070), which is demonstrated in Table 2. The two groups did not have significant differences in sleep time and wake time apart from night awakening minutes (*p* = 0.009) and wake-up time on weekdays (*p* < 0.001).

**Table 2:** CSHQ total scores and subscale scores.

	**Autism patients (*n* = 135)**	**Non-autistic (*n* = 102)**	**T**	***p***
CSHQ mean total score	46.74 ± 9.16	40.86 ± 5.24	6.2^*^	< 0.001^*^
CSHQ bedtime resistance	10.69 ± 3.74	8.3 ± 2.45	5.78^*^	< 0.001^*^
CSHQ sleep onset delay	1.60 ± 0.73	1.39 ± 0.54	2.66^*^	0.008^*^
CSHQ sleep duration	4.17 ± 1.48	4.15 ± 1.34	1.82	0.070
CSHQ sleep anxiety	6.79 ± 2.38	5.45 ± 1.86	5.01^*^	< 0.001^*^
CSHQ night wakings	3.56 ± 1.13	3.22 ± 0.70	2.92^*^	0.004^*^
CSHQ parasomnias	8.83 ± 1.81	7.92 ± 1.21	4.71^*^	< 0.001^*^
CSHQ sleep disordered breathing	3.56 ± 0.99	3.25 ± 0.61	2.94^*^	0.004^*^
CSHQ daytime sleepiness	11.27 ± 4.45	10.10 ± 2.90	2.51^*^	0.013^*^
Weekday bedtime (h:m)	21:51 ± 0:45	21:38 ± 2:15	0.95	0.345
Weekend bedtime	20:42 ± 5:56	20:50 ± 5:37	−0.18	0.861
Weekday amount of sleep	8:54 ± 0:52	8:42 ± 0:54	1.75	0.082
Weekend amount of sleep	9:34 ± 0:57	9:36 ± 1:50	0.17	0.868
Night waking minutes	2.50 ± 8.51	0.46 ± 1.88	2.66^*^	0.009^*^
Weekday wake time	7:10 ± 0:48	6:45 ± 0:37	4.25^*^	< 0.001^*^
Weekend wake time	8:36 ± 1:04	8:45 ± 1:01	−1.09	0.276

Data are presented as mean ± SD.

^*^A p-value of < 0.05.

### 3.3. Factors affecting sleep problems in children with autism

#### 3.3.1. Demographics

**Figure 2:**
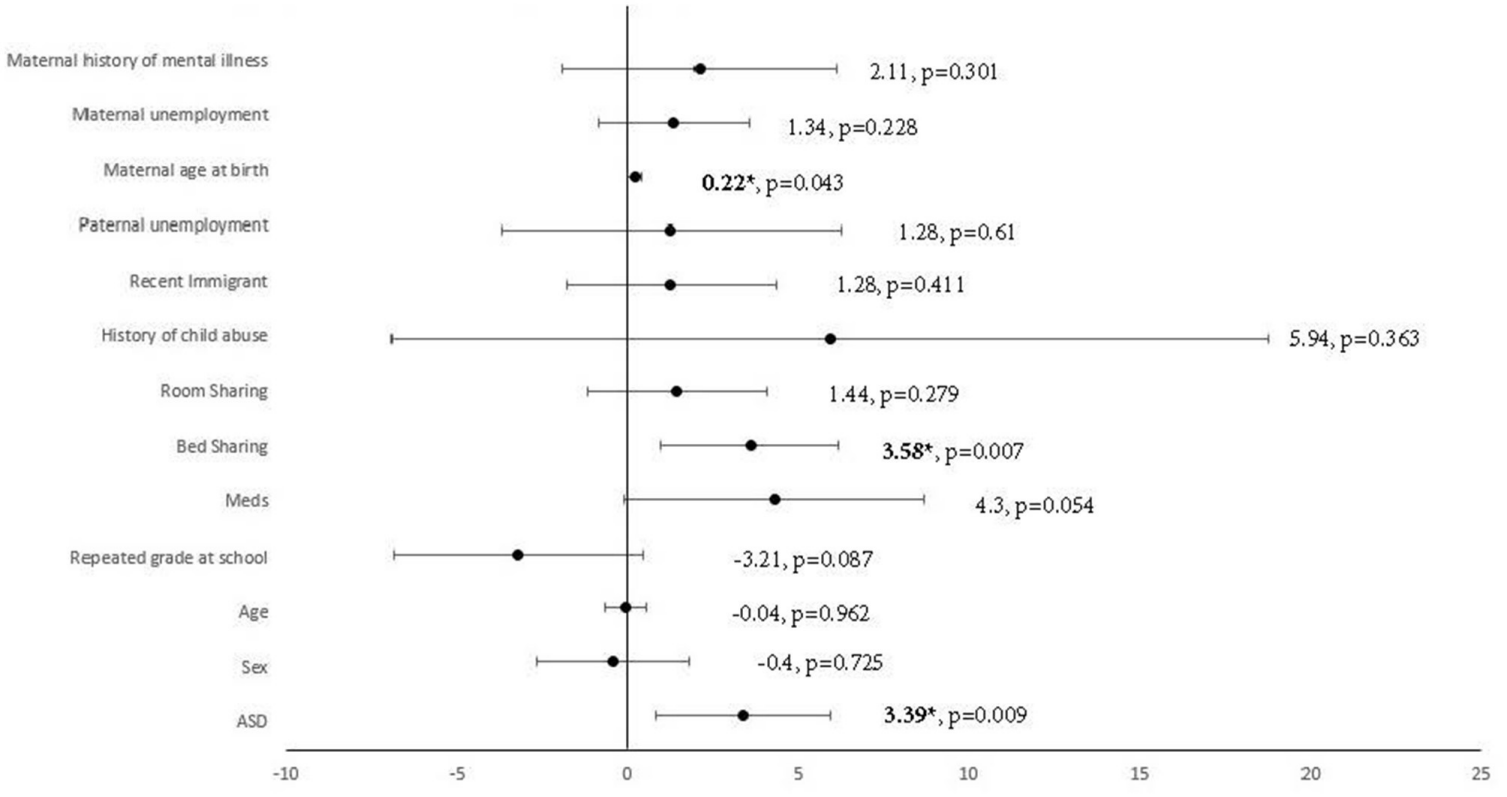
Multiple linear regression analysis using ASD to predict CSHQ total score, adjusted for demographics factors with significant group differences. ^*^Significant after adjustment of *p*-value using Benjamin–Hochberg procedure, with a false discovery rate of 10%. Meds, medication; ASD, autism spectrum disorder.

Multiple linear regression was carried out to investigate whether autism traits could significantly predict the CSHQ total score, adjusted for demographic factors with significant group differences. A significant regression equation was found [*F*_(13, 152)_ = 4.83, *p* < 0.001, *R*^2^ = 0.29]. Autism traits were found to significantly predict the CSHQ total score after adjusting for demographic factors with significant group differences [beta = 0.24, *t*_(165)_ = 2.64, *p* = 0.09], shown in Figure 2. Among the demographic factors, bed-sharing [beta = 0.25, *t*_(165)_ = 2.75, *p* = 0.07] and maternal age at birth [beta = 0.15, *t*_(165)_ = 2.05, *p* = 0.43] were also found to be significant predictors of the CSHQ total score. Pearson correlation was performed to explore the correlations between bed-sharing and all other demographic factors. Bed-sharing showed a significant positive correlation with a maternal history of mental illness [rs_(196)_ = 0.17, *p* = 0.018]. No other demographic factors had a significant correlation with bed-sharing.

#### 3.3.2. Autism features

The Spearman's correlation results showed that sleep problems were positively correlated with the severity of all five domains of autism features, as measured by 3Di (Table 3). The CSHQ total score was significantly correlated with reciprocal social interaction skills (*r* = 0.30, *p* < 0.001), social expressiveness (*r* = 0.17, *p* = 0.045), use of language and other social communication skills (*r* = 0.28, *p* = 0.001), use of gesture and non-verbal play (*r* = 0.20, *p* = 0.019), and repetitive/stereotyped behaviors and routines (*r* = 0.35, *p* = 0.003).

**Table 3:** Spearman's correlation coefficients between 3di subcategory scores and CSHQ total scores.

	**Correlation coefficients with CSHQ total score**	***p***
Reciprocal social interaction skills	0.30	< 0.001^*^
Social expressiveness	0.17	0.045^*^
Use of language and other social communication skills	0.28	0.001^*^
Use of gesture and non-verbal play	0.20	0.019^*^
Repetitive/stereotyped behaviors and routines	0.35	0.003^*^

^*^Indicates significant (p < 0.05).

#### 3.3.3. Co-occurring psychiatric conditions

The Spearman's correlation indicated that there was a significant positive correlation between the number of co-occurring psychiatric conditions and the CSHQ total score (*r* = 0.35, *p* < 0.001). After adjusting for bed -sharing and maternal age at birth, with the *p*-value adjusted using the Benjamin–Hochberg procedure and a false discovery rate of 10%, the stepwise linear regression modeling showed that only separation anxiety disorder (beta = 4.83, *t* = 2.40, *p* = 0.019) predicted the CSHQ score in children with autism (Table 4). For the non-autistic group, no significant difference in CSHQ score between those with or without psychiatric conditions other than autism.

**Table 4:** Regression analysis using co-occurring psychiatric conditions to predict CSHQ total score.

**Co-occurring psychiatric conditions**	**B**	**95% CI**		**Beta**	***t***	***p***
		**Lower bound**	**Upper bound**			
Agoraphobia	−6.23	−17.04	4.59	−0.11	−1.14	0.256
Attention deficit hyperactivity disorder	2.91	−0.08	5.9	0.17	1.94	0.056
Generalized Anxiety Disorder	−4.8	−22	12.36	−0.05	−0.5	0.579
Obsessive compulsive disorder	2.84	−6.46	12.14	0.06	0.61	0.546
Selective mutism	2.15	−12.4	16.7	0.03	0.29	0.77
Separation anxiety disorder	4.83	0.82	8.83	0.23	2.4	0.019^*^
Social phobia	1.31	−3.17	5.79	0.06	0.58	0.564
Specific phobia	1.5	−1.66	4.66	0.09	0.95	0.347

Adjusted for bed-sharing and maternal age at birth.

^*^Indicates significance after adjustment of a p-value using the Benjamin–Hochberg procedure, with a false discovery rate of 10%.

## 4. Discussion

This is the first study in Hong Kong to perform a cross-sectional study on school-age children with autism with a representative community non-autistic group to demonstrate the significant sleep problems in autism and its associated factors.

This study showed that children with diagnosed ASD had more severe sleep problems than healthy children. Particularly, bed-sharing and maternal age at birth contributed to sleep problems in children with autism. The sleep problems were positively correlated with the severity of autism clinical characteristics. Among the co-occurring psychiatric conditions, only separation anxiety disorder has a significant effect on sleep problems in autistic children. These findings highlighted that sleep disturbance is highly prevalent in the autistic population in Hong Kong.

In the current study, the 72% prevalence of sleep problems in autistic children was comparable to other western and local studies where sleep problems ranged between 52 and 86% (8, 9, 45). In agreement with existing studies, bed-sharing with parents was associated with more severe sleep disturbances (25, 46). However, the smaller living environment and perceived family closeness helped to explain the higher prevalence of bed-sharing in Asian than in western cultures (47–50). Up to 81% of parents reported that bed-sharing with children was unplanned and unwanted (51). Although that could be due to children's sleep problems, maternal anxiety, and depression across different ethnicities were also positively associated with bed -sharing (52, 53). As most studies were cross-sectional, further research is needed to establish the causative relationship between bed-sharing and children's sleep disturbance (54).

Older maternal age at birth was found to be associated with increased sleep problems in autistic children. One possible contribution may be the increased risk of offspring with autism for older mothers (20, 30). Yet, data on the direct effect of maternal age at birth on autistic children's sleep was limited (55), further research is suggested to provide further insight into the matter.

In addition, parental employment status in this study also showed group differences. Previous studies in Australia and the United States have shown that parental employment status is impacted by the childcare of children with autism (56, 57). Parents of children with autism are more likely to be unemployed, especially the maternal side. Household income and average working hours are also negatively impacted. Studies in China also demonstrated that childcare for autistic children impacted employment by an odds ratio of 15.94, while there was a significant loss of household income by about 50% (58). As aforementioned, one of the main contributors to bed-sharing was maternal anxiety, which may explain the significant correlation between the two.

Findings identified that the severity of sleep problems was positively correlated with the severity of autism traits. Consistent with previous literature, increased social impairment and stereotypic behavior were associated with greater sleep problems in children with autism (13, 59). Clock gene variations were postulated to be a reason for the circadian cycle disruption seen in autistic children, while gastrointestinal disturbances were also prominent in children with autism (60). Such observation may suggest that the severity of sleep disturbances and autism traits reflect the degree of genetic variation in the child, which may be a direction for future research.

Finally, the association between separation anxiety disorder in sleep problems and insistence on sameness, mediated by sensory hypersensitivity, was only found in autistic children (61). While data concerning treatment for autistic children with co-occurring separation anxiety disorder is limited, further research concerning treatment modalities for a separation anxiety disorder should also look into the treatment effects on sleep problems. In addition, it is worth noting that the co-occurring relationship between ADHD and ASD in the prediction of sleep problems was only marginally significant (*p* = 0.056), thus, a larger sample may reflect a different result. Previous studies have shown that ADHD is a common co-occurring psychiatric condition of ASD and that the similar sleep disturbance found in both groups helped to explain the greater internalizing and externalizing behavioral problems in children with co-occurring psychiatric conditions (62, 63).

There are some limitations in the current study. Despite CSHQ having high sensitivity to detect sleep problems, only using this standardized parent-report questionnaire could not indicate the specific pattern of sleep disturbance. Moreover, other factors such as diet, and ongoing treatment types were not controlled for. Since the current study included a scattered sample of income, education, and immigration status, the representativeness of the findings may be limited. Future studies may consider using actigraphy or PSG to identify a specific pattern of sleep disturbance in children with autism. Due to the cross-sectional nature of the current study, the overtime impact of sleep problems was not investigated. Further follow-up studies could provide insights into the sleep problems' relationship with time, and possibly the effect of treatment on such over time.

## 5. Clinical implications and conclusion

The findings in the present study have an impact on the clinical management and research on autism. Given the high prevalence and the impact of sleep problems, clinicians need to take a detailed sleep history to rule out any sleep problems, particularly in children with older mothers. CSHQ can be used in a usual clinic setting to identify possible sleep problems, or provided during triage. During the consultation, parental education regarding the possible role of bed-sharing to sleep problems should be provided upon identification of bed-sharing. Clinics can also adopt structured sleep behavioral interventions to help parents provide a proper sleeping routine, prescribe medications in consideration of sleep problems, and provide psychoeducation and cognitive–behavioral techniques to parents of autistic children with co-occurring separation anxiety disorder.

While our study was able to identify increased sleep disturbances in autistic children through questionnaires, future research can focus on other more objective assessment tools, such as actigraphy and PSG. A follow-up study can help to identify modifiable factors that would improve sleep problems in long term. Our study was also able to highlight the heterogenous yet controversial factor of bed -sharing. Future research on bed-sharing can explore the reasons and proportion of unwanted bed-sharing by parents in Hong Kong culture, which may also exist in other crowded cities in the world. Finally, future study is also advised to explore the impact and possible factors including demographics (i.e., parental age and immigration status) on sleep quality, which could serve as a potential intervention area in parental education.

## Data availability statement

The raw data supporting the conclusions of this article will be made available by the authors, without undue reservation.

## Ethics statement

The studies involving human participants were reviewed and approved by the Institutional Review Board of the University of Hong Kong/Hospital Authority Hong Kong West Cluster (reference: UW 19-290). Written informed consent to participate in this study was provided by the participants' legal guardian/next of kin.

## Author contributions

All authors listed have made a substantial, direct, and intellectual contribution to the work and approved it for publication.
